# Variations in coil temperature/power and e‐liquid constituents change size and lung deposition of particles emitted by an electronic cigarette

**DOI:** 10.14814/phy2.14093

**Published:** 2019-05-29

**Authors:** Ariane Lechasseur, Simon Altmejd, Natalie Turgeon, Giorgio Buonanno, Lidia Morawska, David Brunet, Caroline Duchaine, Mathieu C. Morissette

**Affiliations:** ^1^ Quebec Heart and Lung Institute ‐ Université Laval Quebec Quebec Canada; ^2^ Faculty of Medicine Université Laval Quebec Quebec Canada; ^3^ SCIREQ Scientific Respiratory Equipment Inc. Montreal Canada; ^4^ University of Cassino and Southern Lazio Cassino Italy; ^5^ Queensland University of Technology Brisbane Australia; ^6^ Departement of Biochemistry, Microbiology and Bioinformatics Université Laval Quebec Quebec Canada; ^7^ Department de Medicine Université Laval Quebec Quebec Canada

**Keywords:** Electronic cigarette, e‐liquid, lung distribution, nicotine, particle size, vaping

## Abstract

Electronic cigarette uses propylene glycol and glycerol to deliver nicotine and flavors to the lungs. Given the hundreds of different brands, the thousands of flavors available and the variations in nicotine concentrations, it is likely that electronic cigarette settings and e‐liquid composition affect the size distribution of particles emitted and ultimately pulmonary deposition. We used the inExpose e‐cigarette extension to study two separate modes of operation of electronic cigarettes, namely power‐controlled and the temperature‐controlled. We also assessed several e‐liquids based on propylene glycol and glycerol concentrations, nicotine content, and selected monomolecular flavoring agents (menthol, vanillin, and maltol). Particle size distribution was measured using a Condensation Particle Counter and a Scanning Mobility Particle Sizer spectrometer. Lung deposition was predicted using the International Commission on Radiological Protection model. For all resistance coils, increase in power delivery generated larger particles while maintaining a higher coil temperature generated smaller particles. Increase in glycerol concentration led to the generation of larger particles. With regard to flavors, we showed that despite minor effect of menthol and maltol, vanillin dramatically increased particle size. Presence of nicotine also increased particle size. Finally, particles emitted by the electronic cigarette were predicted to mainly deposit in the alveoli and conditions generating larger particle sizes led to a reduction in predicted lung deposition. This study shows that coil temperature, propylene glycol and glycerol concentrations, presence of nicotine, and flavors affect the size of particles emitted by an electronic cigarette, directly affecting predicted lung deposition of these particles.

## Introduction

Electronic cigarette (e‐cigarette) use has markedly increased over the past years. A recent study conducted in the United States showed that e‐cigarette use has even surpassed tobacco cigarette use among middle school and high school students (Singh et al. 2016). The market for e‐cigarettes is greatly diversified. In 2017, 433 brands of e‐cigarettes were commercially available (Zhu et al. 2014; Hsu et al. 2018). Moreover, e‐liquids are available in over 7000 flavors, with nicotine concentrations ranging from 0 mg/mL to 24 mg/mL (Goniewicz et al. 2013; Zhu et al. 2014; Hsu et al. 2018).

Upon inhalation, the e‐liquid, a mixture of propylene glycol (PG), glycerol (Gly), nicotine and/or flavors, is dragged through a heating coil, which leads to its aerosolization (Brown and Cheng 2014; Talih et al. 2017). The popularity of e‐cigarettes is mainly due to the impression of safety surrounding its use (Camenga et al. 2015; Farsalinos et al. 2015; Majeed et al. 2017). However, studies have shown that e‐cigarette vapors contain several oxidants, carcinogens and irritants, such as formaldehyde, acetaldehyde, acrolein, methylglyoxal, and other free radicals (Bekki et al. 2014; Margham et al. 2016; Farsalinos et al. 2017). It has been shown that increasing puff duration and coil power can increase the generation of these hazardous components (Gillman et al. 2016; Farsalinos et al. 2017). Moreover, addition of nicotine and flavorings also increases the number of potential irritants that are inhaled (Khlystov and Samburova 2016; Bitzer et al. 2018). However, we currently do not know how e‐cigarette settings and e‐liquid constituents specifically impact the size of the particle generated and, consequently, lung deposition.

Particle aerodynamic diameter is the main predictor of where inhaled particles will deposit into the lungs and in what proportion (ICRP, 1994). Since variations in aerosolization conditions can very likely impact particle size and ultimately lung deposition, it is critical to assess the impact of the multiple product variation of e‐cigarettes (i.e. coil power, PG/Gly ratios, flavors, nicotine content) on particle size and determine how it affects lung deposition. In this study, we assessed the impact of power, temperature, PG/Gly ratios, flavors, and nicotine content on the size of particles emitted by an e‐cigarette using a single brand of e‐cigarette and found that all modifiable aspects of e‐cigarette settings tested or e‐liquid constituents directly affect particle size and lung deposition.

## Methods

### Electronic cigarette and aerosol generation

The inExpose e‐cigarette extension (SCIREQ, Montreal, PQ) was used in this study. The inExpose e‐cigarette extension is composed of a *Joyetech eVIC‐VTC Mini* e‐cigarette connected to a computer‐controlled system that automates the e‐cigarette activation and standardizes the vaping conditions for research purposes. The inExpose system bypasses the native battery of the *eVIC‐VTC,* thereby eliminating aerosol output variations associated with battery drainage. The inExpose puff profiles were configured to a half‐sinusoidal shape with a volume of 70 mL, applied every 30 sec. Total puff run time was of 4.2 sec. The inExpose system also provided a 2 LPM bias flow to push the e‐cigarette vapor into the 45 L dilution chamber.

The *eVIC‐VTC* can be configured in two distinct modes: power‐controlled and temperature‐controlled. When configured in power‐controlled mode, a preset power value is selected by the user (0.5 Ω range 15 W–60 W; 1.5 Ω range: 10–25 W). During a puff, the power delivered to the coil stays relatively constant during the puff cycle. The constant power translates into a steady increase of the coil's temperature throughout the puff cycle. In the temperature‐controlled mode, the power transferred to the coil is regulated with a feedback mechanism. This closed‐loop control aims to maintain a constant temperature throughout the puff. The temperature set point is adjustable (range 200–250°C) and configured by the user.

During experiments under the power‐controlled mode (50% PG/50% Gly ratio), two different stainless steel coils were used with respective resistance of 0.5 Ω and 1.5 Ω. Each of these coils was tested at three different power levels (0.5 Ω coil at 24, 37.5 and 51 W and 1.5 Ω coil at 13.2, 18 and 22.8 W). Experiments using the temperature‐controlled mode (50% PG/50% Gly ratio) were also conducted. The temperature‐controlled experiments were carried out with a coil made of nickel, using the following set points: 210, 225 and 250°C. To assess the impact of PG/Gly ratios, flavors and nicotine, the temperature‐controlled setting was used at 210°C. The e‐liquids used in the study were composed of 100% PG/0% Gly, 70% PG/30% Gly, 30% PG/70% Gly or 0% PG/100% Gly, with or without 18 mg/mL of nicotine. Menthol (10 mg/mL), vanillin (10 mg/mL) or maltol (5 mg/mL) were added to a 70% PG/30% Gly or 30% PG/70% Gly e‐liquid, in concentrations based on previous studies (Tierney et al. 2016). Flavors and nicotine were added to a 70% PG/30% Gly e‐liquid.

### Instrumentation and aerosol sampling

A new heating coil was used for each parameter investigated. To avoid dry puffing, five puffs (70 mL puff, 2 per minute) were made outside the collection system. A total of five puffs (70 mL puff, 2 per minute with a 2 L/min bias flow) were generated and collected. Vapors diluted with 40 L/min airflow were collected in a 45 L barrel placed in a biosafety cabinet to avoid room air particles from being sampled. Measures for each experimental condition were performed in triplicate.

### E‐cigarette particle size distribution analyses

Measurements of particle size distribution were carried out by a Condensation Particle Counter (CPC 3787, TSI Inc.) and a Scanning Mobility Particle Sizer spectrometer (SMPS 3080, TSI Inc). Particle size range was fixed at 20.9 to 881.7 nm. Data collection was performed during a scan time of 120 sec with a sheath flow of 2 LPM and an aerosol flow of 0.2 LPM. Each data acquisition was made in triplicate. Each curve represents the proportion of each particle diameter normalized to the total number of particles analyzed (% of total particles analyzed).

### E‐cigarette particle lung deposition analyses

Lung deposition was calculated using the International Commission on Radiology Protection model (ICRP). Total, head airway region, tracheobronchial airway region, and alveolar airway region deposition were assessed according to previously published work (ICRP, 1994):

The head airway deposition fraction DF_HA_ is

$${\mathrm{DF}}_{\mathrm{HA}}=\mathrm{IF}\left(\frac{1}{1+e^{6.84+1.183\ln\;d_{p}}}+\frac{1}{1+e^{0.924-1.885\ln\;d_{p}}}\right)$$

where d_p_ is the particle size in μm and IF is the inhalable fraction, given by

$$\mathrm{IF}=1-0.5\left(1-\frac{1}{1+0.00076d_{p}^{2.8}}\right)$$

The tracheobronchial deposition fraction DF_TB_ is

$$\begin{array}{cc} & {\mathrm{DF}}_{\mathrm{TB}}=\left(\frac{0.00352}{d_{p}}\right)\\ & \left(e^{-0.234{\left(\ln\;d_{p}+3.40\right)}^{2}}+63.9e^{-0.819{\left(\ln\;d_{p}-1.61\right)}^{2}}\right) \end{array}$$

The alveolar deposition fraction DF_AL_ is

$$\begin{array}{cc} & {\mathrm{DF}}_{\mathrm{AL}}=\left(\frac{0.0155}{d_{p}}\right)\\ & \left(e^{-0.416{\left(\ln\;d_{p}+2.84\right)}^{2}}+19.11e^{-0.482{\left(\ln\;d_{p}-1.392\right)}^{2}}\right) \end{array}$$

The total deposition DF is the sum of the regional depositions, or

$$\mathrm{DF}=\mathrm{IF}\left(0.0587+\frac{0.911}{1+e^{4.77+1.485\ln\;d_{p}}}\right)+\left(\frac{0.943}{1+e^{0.508-2.58\ln\;d_{p}}}\right)$$

Each curve represents the deposition in each lung region multiplied by the emitted relative proportion of each particle diameter analyzed.

### Statistical analyses

Particle size distribution between two experimental groups was assessed by a Kolmogorov–Smirnov test (Table S1). Lung particle deposition between two experimental groups was also assessed using a Kolmogorov–Smirnov test (Table S2). Resulting *P*‐values are indicated in Tables S1 and S2, a *P*‐value <0.05 indicating a significantly different distribution between the two compared groups. Statistical analyses were made using GraphPad Prism Software (v. 8, La Jolla California USA).

## Results

### E‐cigarette particle size increases in a coil power‐dependent manner

**Figure 1:**
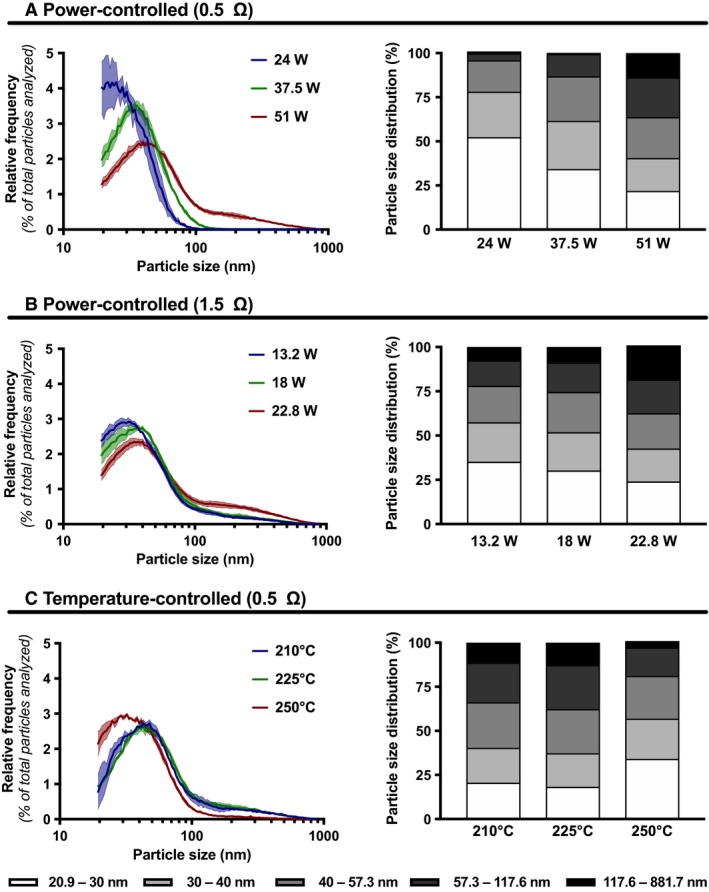
Impact of coil power and temperature on size distribution of particles emitted by an e‐cigarette. Size distribution and size intervals of particles emitted by an e‐cigarette under (A) a power‐controlled setting with a 0.5 Ω coil, (B) a power‐controlled setting with a 1.5 Ω coil, and (C) a temperature‐controlled setting with a 0.5 Ω coil. In all cases, a 50% PG/50% Gly e‐liquid ratio was used, with no nicotine or flavors. Mean (hard line) of three replicates per condition ± standard error mean (shade). For each replicate, particle diameter frequencies were normalized to the total number of particles analyzed.

A large variety of e‐cigarette brands are commercially available, meaning numerous possible combinations in coil resistance, power settings, and temperature. We first assessed the impact of the coil power on the e‐cigarette particle size distribution. A 50% PG/50% Gly e‐liquid without flavors or nicotine was used. For the 0.5 Ω coil, we found that increased coil power led to the generation of larger particles (Fig. 1A; Table S1). Similar trends were found for the 1.5 Ω coil (Fig. 1B; Table S1). Intriguingly, while the two lowest temperatures of the temperature‐controlled mode generated similar particle size distribution, smaller particles were emitted while using the highest temperature setting (Fig. 1C; Table S1).

### A greater proportion in e‐liquid glycerol leads to larger e‐cigarette particle size

**Figure 2:**
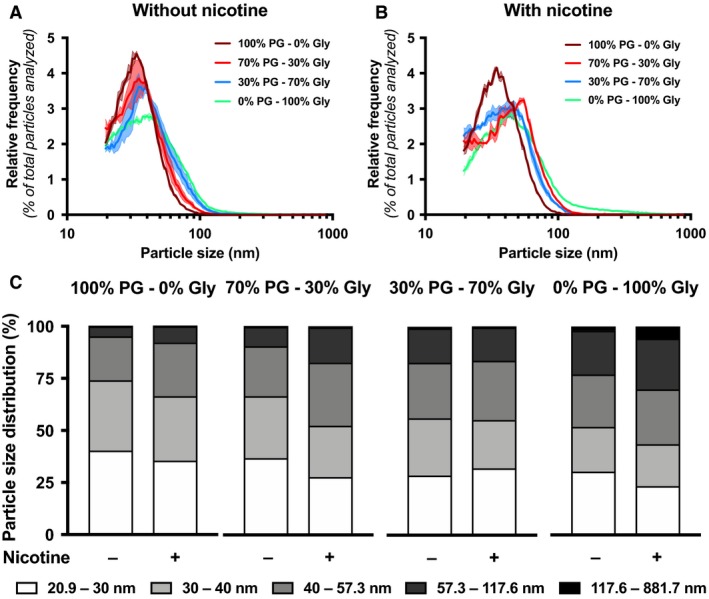
Impact of PG/Gly ratios and nicotine on size distribution of particles emitted by an e‐cigarette. Size distribution of particles emitted by an e‐cigarette under temperature‐controlled set at 210°C with e‐liquid containing (A) 0 mg/mL of nicotine or (B) 18 mg/mL of nicotine. In both cases, e‐liquids made of 100% PG/0% Gly (maroon line), 70% PG/30% Gly (red line), 30% PG/70% Gly (blue line) or 0% PG/100% Gly (teal line) were used, all without flavors. (C) Size intervals of particles emitted are presented. Mean (hard or dotted lines) of three replicates per condition ± standard error mean (shade). For each replicate, particle diameter frequencies were normalized to the total number of particles analyzed.

**Figure 3:**
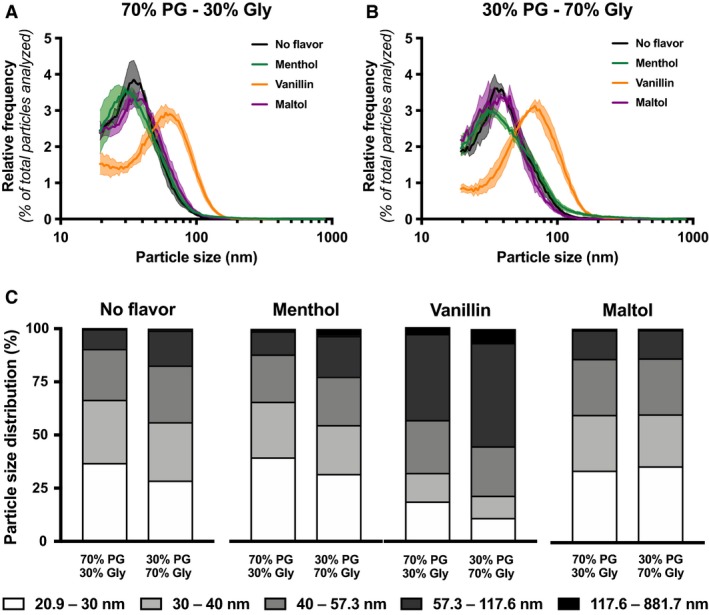
Impact of menthol, vanillin, or maltol on size distribution of particles emitted by an e‐cigarette. Size distribution of particles emitted by an e‐cigarette under temperature‐controlled set at 210°C with (A) 70% PG/30% e‐liquid or (B) 30% PG/70% Gly e‐liquid containing no flavor (black line), menthol (green line), vanillin (orange line) or maltol (purple line). (C) Size intervals of particles emitted are presented. Mean (hard lines) of three replicates per condition ± standard error mean (shade). For each replicate, particle diameter frequencies were normalized to the total number of particles analyzed.

We investigated the impact of different PG/Gly ratios on the e‐cigarette particle emission. We found that higher Gly proportion, with and without nicotine, led to the generation of larger particles (Fig. 2A–C; Table S1). This phenomenon was also shown in menthol and vanillin containing e‐liquids, as larger particles were generated in flavor‐containing 30% PG/70% Gly e‐liquid compared to the 70% PG/30% Gly e‐liquid (Fig. 3A–C; Table S1).

### Nicotine changes e‐cigarette particle size distribution

**Figure 4:**
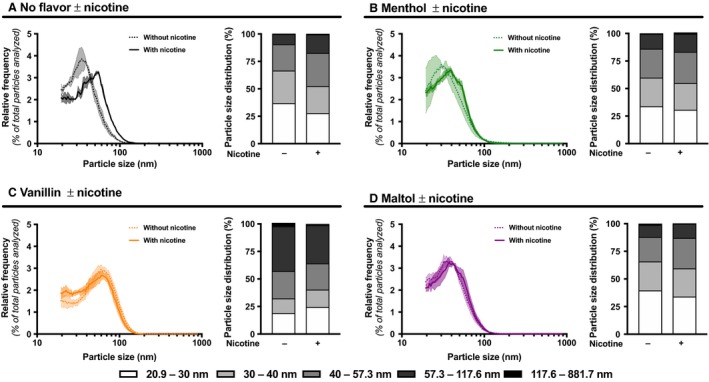
Impact of nicotine with menthol, vanillin, or maltol on size distribution of particles emitted by an e‐cigarette. Size distribution and size intervals of particles emitted by an e‐cigarette under temperature‐controlled set at 210°C with 70% PG/30% Gly e‐liquid with (A) no flavor, (B) menthol (C), vanillin or (D) maltol without nicotine (dotted line) or with 18 mg/mL of nicotine (hard line). Mean (lines) of three replicates per condition ± standard error mean (shade). For each replicate, particle diameter frequencies were normalized to the total number of particles analyzed. For comparisons purposes, Figure 4 presents controls without nicotine that are also presented in Figure 3A.

Addition of nicotine in e‐liquids is very common, with concentrations ranging from 0 to 24 mg/mL (Tierney et al. 2016). We therefore assessed the impact of nicotine on e‐cigarette particle size distribution. Regardless of PG/Gly ratios, addition of nicotine to flavor‐free e‐liquid increased emitted particle size (Fig. 2A–C; Table S1). However, adding nicotine to flavored e‐liquid (menthol, vanillin or maltol) did not affect particle size distribution (Fig. 4A–C; Table S1).

### Vanillin increase e‐cigarette particle size

Several flavonoids are used to reproduce the 7000 flavors in which e‐liquids are sold. We further assessed the impact of flavors on particle size distribution. We found that adding menthol or maltol to the e‐liquid did not change the particle size distribution compared to the unflavored e‐liquid (Fig. 3A–B; Table S1). However, adding vanillin drastically increased the e‐cigarette–emitted particle size (Fig. 3A–B; Table S1).

### Variations in e‐cigarette components and e‐liquid composition affect the predicted lung deposition

**Figure 5:**
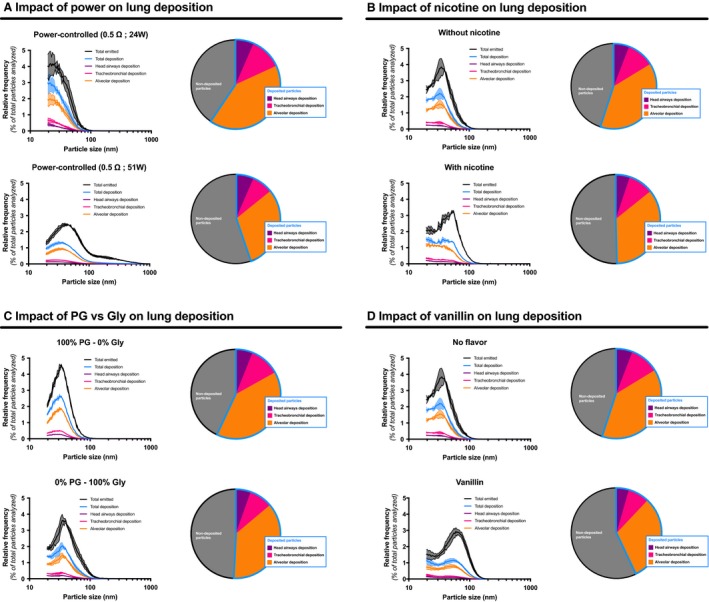
Impact of variations in e‐cigarette settings and e‐liquid constituents on lung deposition of emitted particles. Lung deposition of particles emitted by the e‐cigarette was calculated according to the International Commission on Radiology Protection (ICRP) model. Impact of (A) e‐cigarette power, (B) presence of nicotine in the e‐liquid, (C) PG‐based or Gly‐based e‐liquid and (D) presence of vanillin in the e‐liquid are presented. Mean (hard line) of three replicates per condition ± standard error mean (shade). Each pie chart represents the percentage of total deposited particles that are specifically deposited in the head region (purple section), tracheobronchial region (pink section), and alveolar region (orange section). For each replicate, particle diameter frequencies were normalized to the total number of particles analyzed.

We observed several effects of e‐cigarette settings and e‐liquid constituents on particle size and distribution. Using preestablished lung deposition equations for head airways, tracheobronchial airways, and alveoli, we calculated how variations in particle size distribution affect predicted lung deposition. Particles generated by the e‐cigarette at any setting and with any e‐liquid were predicted to mainly deposit in the alveoli. Conditions that led to an increase in particle size generated by the e‐cigarette, such as increase in power and e‐liquid glycerol proportion as well as presence of nicotine and vanillin in the e‐liquid, led to a reduction in alveolar deposition (Fig. 5; Table S2).

## Discussion

This study is one of the first to document that changing e‐cigarette settings and e‐liquid composition has an impact on particle size distribution. Consequently, this variation in particle size is also predicted to change how particles emitted by the e‐cigarette deposit in the lungs.

In this study, we were able to modulate the power of the heating coil using a single e‐cigarette brand. Under the power‐controlled setting, we have shown that increased heating coil power leads to increased particle size. This phenomenon was not reproduced when using the temperature‐controlled setting. This could be explained by the fact that, since having a fixed power instead of a fixed endpoint temperature, the power‐controlled coil reaches greater temperatures than the temperature‐controlled coil. Gillman et al. (2016) assessed the difference between different brands of e‐cigarette, showing that greater coil power led to the generation of greater e‐cigarette aerosol mass and formaldehyde, acetaldehyde, and acrolein levels. This shows that changes in e‐cigarette model, and therefore coil power, can not only change the particle size distribution but can also change the composition of the aerosols that will be delivered to the lungs.

E‐liquids can be sold in several PG/Gly ratios and in a wide range of nicotine concentrations. We found that PG/Gly ratio can impact particle size distribution, as higher Gly concentration increases particle size, as has previously been observed in other studies (Baassiri et al. 2017; Larcombe et al. 2017). This phenomenon could be explained by the fact that PG has a higher volatility than Gly (NCBI, 2018a,b). Upon heating, PG is aerosolized at a lower temperature, and thus faster than Gly. Until Gly reaches its aerosolization temperature, condensation is formed in the e‐cigarette, leading in time to the formation of larger particles. Real‐time assessment of e‐cigarette aerosol particle composition and size distribution could help elucidate this phenomenon.

A wide range of nicotine concentrations and flavors in e‐liquids are available on the market. We showed that the addition of nicotine also increases particle size. Others have shown that addition of nicotine increases the number of particles that are generated (Fuoco et al. 2014; Manigrasso et al. 2015), as well as their size (Larcombe et al. 2017; Laube et al. 2017). Although we do not fully understand this phenomenon, it appears consistent across experimental settings that presence of nicotine leads to higher numbers and larger particles and, consequently, a reduced lung deposition.

We also assessed the impact of adding laboratory grade menthol, maltol and vanillin, three aromatic molecules frequently found in e‐liquids (Tierney et al. 2016), on e‐cigarette particle size. In their study, Fuoco et al. (2014) did not report any changes in particle size when using selene‐flavored, strawberry‐flavored or two different tobacco‐flavored e‐liquids. Here, we show that, while menthol and maltol had mild impact on particle size, the addition of vanillin increased particle size. This shows that flavors can have different effects on particle size and that findings made using a given flavor cannot be easily extrapolated to another. Chemical properties of certain flavor molecules could facilitate the generation of larger particle compared to others. However, this remains to be confirmed experimentally.

Changes in power, PG/Gly ratios, nicotine concentration, and flavors can change the e‐cigarette emitted particle size distribution, potentially affecting lung deposition. Using the ICRP deposition model, we estimated how changes in particle size affected lung deposition (Fig. 5; Table S2). Changes were observed in the total deposition fraction by changes in coil power (Fig. 5A; Table S2), nicotine concentration (Fig. 5B; Table S2), PG/Gly ratios (Fig. 5C; Table S2), and the addition of vanillin (Fig. 5D; Table S2). While few changes in deposition were observed in the head airway region and tracheobronchial airway region, drastic changes in alveolar airway deposition were observed in each variable analyzed. For e‐cigarette users, these differences suggest changes in the nicotine deposition, as well as the lung deposition of aforementioned harmful chemical compounds such as formaldehyde, acetaldehyde, acrolein, and other free radicals.

Overall, this study shows that changing the e‐cigarette setting and e‐liquid composition can alter e‐cigarette particle size distribution, leading to changes in lung deposition. This may affect the amount of nicotine that is absorbed, and how much PG/Gly and flavors interact with the alveoli. It also highlights how flavoring agents can drastically alter the physicochemical nature of e‐liquids. The physiological impacts of these changes remain to be investigated.

## Conflict of Interest

None declared.

## Supporting information




**Figure S1.** Pictures of the vapor‐generating device, dilution drum and particle analysing system.
**Table S1.** Statistical analysis of the impact of electronic cigarette settings and e‐liquid constituents on particle size distribution.
**Table S2.** Statistical analysis of the impact of electronic cigarette settings and e‐liquid constituents on predicted lung deposition of aerosolized particles.

## References

[phy214093-bib-0001] Baassiri, M. , S. Talih , R. Salman , N. Karaoghlanian , R. Saleh , R. el Hage , et al. 2017 Clouds and “throat hit”: effects of liquid composition on nicotine emissions and physical characteristics of electronic cigarette aerosols. Aerosol Sci. Technol. 51:1231–1239. 10.1080/02786826.2017.1341040 PMC7453347 32863527

[phy214093-bib-0002] Bekki, K. , S. Uchiyama , K. Ohta , Y. Inaba , H. Nakagome , and N. Kunugita . 2014 Carbonyl compounds generated from electronic cigarettes. Int. J. Environ. Res. Public Health 11:11192–11200. 25353061 10.3390/ijerph111111192 PMC4245608

[phy214093-bib-0003] Bitzer, Z. T. , R. Goel , S. M. Reilly , R. J. Elias , A. Silakov , J. Foulds , et al. 2018 Effect of flavoring chemicals on free radical formation in electronic cigarette aerosols. Free Radic. Biol. Med. 120:72–79. 29548792 10.1016/j.freeradbiomed.2018.03.020 PMC5940571

[phy214093-bib-0004] Brown, C. J. , and J. M. Cheng . 2014 Electronic cigarettes: product characterisation and design considerations. Tob. Control 23(Suppl 2):ii4–Ii10. 24732162 10.1136/tobaccocontrol-2013-051476 PMC3995271

[phy214093-bib-0005] Camenga, D. R. , D. A. Cavallo , G. Kong , M. E. Morean , C. M. Connell , P. Simon , et al. 2015 Adolescents’ and young adults’ perceptions of electronic cigarettes for smoking cessation: a focus group study. Nicotine Tob. Res. 17:1235–1241. 25646346 10.1093/ntr/ntv020 PMC4607731

[phy214093-bib-0006] Farsalinos, K. E. , G. Romagna , and V. Voudris . 2015 Factors associated with dual use of tobacco and electronic cigarettes: a case control study. Int. J. Drug Policy 26:595–600. 25687714 10.1016/j.drugpo.2015.01.006

[phy214093-bib-0007] Farsalinos, K. E. , V. Voudris , A. Spyrou , and K. Poulas . 2017 E‐cigarettes emit very high formaldehyde levels only in conditions that are aversive to users: a replication study under verified realistic use conditions. Food Chem. Toxicol. 109:90–94. 28864295 10.1016/j.fct.2017.08.044

[phy214093-bib-0008] Fuoco, F. C. , G. Buonanno , L. Stabile , and P. Vigo . 2014 Influential parameters on particle concentration and size distribution in the mainstream of e‐cigarettes. Environ. Pollut. 184:523–529. 24172659 10.1016/j.envpol.2013.10.010

[phy214093-bib-0009] Gillman, I. G. , K. A. Kistler , E. W. Stewart , and A. R. Paolantonio . 2016 Effect of variable power levels on the yield of total aerosol mass and formation of aldehydes in e‐cigarette aerosols. Regul. Toxicol. Pharmacol. 75:58–65. 26743740 10.1016/j.yrtph.2015.12.019

[phy214093-bib-0010] Goniewicz, M. L. , T. Kuma , M. Gawron , J. Knysak , and L. Kosmider . 2013 Nicotine levels in electronic cigarettes. Nicotine Tob. Res. 15:158–166. 22529223 10.1093/ntr/nts103

[phy214093-bib-0011] Hsu, G. , J. Y. Sun , and S. H. Zhu . 2018 Evolution of electronic cigarette brands from 2013‐2014 to 2016‐2017: analysis of brand websites. J. Med. Internet Res. 20:e80. 29530840 10.2196/jmir.8550 PMC5869180

[phy214093-bib-0012] ICRP . 1994 Human respiratory tract model for radiological protection. Annals of the ICRP. International Commission on Radiology Protection. 7726471

[phy214093-bib-0013] Khlystov, A. , and V. Samburova . 2016 Flavoring compounds dominate toxic aldehyde production during E‐cigarette vaping. Environ. Sci. Technol. 50:13080–13085. 27934275 10.1021/acs.est.6b05145

[phy214093-bib-0014] Larcombe, A. N. , M. A. Janka , B. J. Mullins , L. J. Berry , A. Bredin , and P. J. Franklin . 2017 The effects of electronic cigarette aerosol exposure on inflammation and lung function in mice. Am. J. Physiol. Lung Cell. Mol. Physiol. 313:L67–L79. 28360111 10.1152/ajplung.00203.2016

[phy214093-bib-0015] Laube, B. L. , N. Afshar‐Mohajer , K. Koehler , G. Chen , P. Lazarus , J. M. Collaco , et al. 2017 Acute and chronic in vivo effects of exposure to nicotine and propylene glycol from an E‐cigarette on mucociliary clearance in a murine model. Inhal. Toxicol. 29:197–205. 28651446 10.1080/08958378.2017.1336585 PMC5553614

[phy214093-bib-0016] Majeed, B. A. , S. R. Weaver , K. R. Gregory , C. F. Whitney , P. Slovic , T. F. Pechacek , et al. 2017 Changing perceptions of harm of E‐cigarettes among U.S. adults, 2012‐2015. Am. J. Prev. Med. 52:331–338. 28341303 10.1016/j.amepre.2016.08.039 PMC5373478

[phy214093-bib-0017] Manigrasso, M. , G. Buonanno , F. C. Fuoco , L. Stabile , and P. Avino . 2015 Aerosol deposition doses in the human respiratory tree of electronic cigarette smokers. Environ. Pollut. 196:257–267. 25463721 10.1016/j.envpol.2014.10.013

[phy214093-bib-0018] Margham, J. , K. McAdam , M. Forster , C. Liu , C. Wright , D. Mariner , et al. 2016 Chemical composition of aerosol from an E‐cigarette: a quantitative comparison with cigarette smoke. Chem. Res. Toxicol. 29:1662–1678. 27641760 10.1021/acs.chemrestox.6b00188

[phy214093-bib-0019] NCBI . 2018a PubChem Compound Database; CID = 753 [Online]. Available: https://pubchem.ncbi.nlm.nih.gov/compound/753 [Accessed 29 Oct 2018].

[phy214093-bib-0020] NCBI . 2018b PubChem Compound Database; CID = 1030 [Online]. Available: https://pubchem.ncbi.nlm.nih.gov/compound/1030 [Accessed 29 Oct 2018].

[phy214093-bib-0021] Singh, T. , R. A. Arrazola , C. G. Corey , C. G. Husten , L. J. Neff , D. M. Homa , et al. 2016 Tobacco use among middle and high school students‐United States, 2011–2015. Morb. Mortal. Wkly Rep. 65:361–367. 10.15585/mmwr.mm6514a1 27077789

[phy214093-bib-0022] Talih, S. , Z. Balhas , R. Salman , R. El‐Hage , N. Karaoghlanian , A. El‐Hellani , et al. 2017 Transport phenomena governing nicotine emissions from electronic cigarettes: model formulation and experimental investigation. Aerosol Sci. Technol. 51:1–11. 28706340 10.1080/02786826.2016.1257853 PMC5502764

[phy214093-bib-0023] Tierney, P. A. , C. D. Karpinski , J. E. Brown , W. Luo , and J. F. Pankow . 2016 Flavour chemicals in electronic cigarette fluids. Tob. Control 25:e10–e15. 25877377 10.1136/tobaccocontrol-2014-052175 PMC4853541

[phy214093-bib-0024] Zhu, S. H. , J. Y. Sun , E. Bonnevie , S. E. Cummins , A. Gamst , L. Yin , et al. 2014 Four hundred and sixty brands of e‐cigarettes and counting: implications for product regulation. Tob. Control 23(Suppl 3):iii3–iii9. 24935895 10.1136/tobaccocontrol-2014-051670 PMC4078673

