# Supplementary material for: Variations in coil temperature/power and e‐liquid constituents change size and lung deposition of particles emitted by an electronic cigarette
Source: Physiol Rep. 2019 May 29;7(10):e14093. doi: 10.14814/phy2.14093 (PMC6540444; doi:10.14814/phy2.14093)
Supplement: Supplementary file 1 — Figure S1. Pictures of the vapor‐generating device, dilution drum and particle analysing system. Table S1. Statistical analysis of the impact of electronic cigarette settings and e‐liquid constituents on particle size distribution. Table S2. Statistical analysis of the impact of electronic cigarette settings and e‐liquid constituents on predicted lung deposition of aerosolized particles. [file PHY2-7-e14093-s001.docx]

**SUPPLEMENTARY MATERIAL**

**Variations in coil temperature/power and e-liquid constituents change size and lung deposition of particles emitted by an electronic cigarette**

Ariane Lechasseur^1,2^, Simon Altmejd^3^, Natalie Turgeon^1^, Giorgio Buonanno^4,5^, Lidia Morawska^5^, David Brunet^3^, Caroline Duchaine^1,6^, Mathieu C Morissette^1,7^

^1^Quebec Heart and Lung Institute - Université Laval, ^2^Faculty of Medicine, Université Laval, ^3^SCIREQ Scientific Respiratory Equipment Inc., ^4^University of Cassino and Southern Lazio, Italy, ^5^Queensland University of Technology, Australia, ^6^Department de Medicine, Université Laval, ^7^Departement of biochemistry, microbiology and bioinformatics, Université Laval

**Figure S1. Pictures of the vapour-generating device, dilution drum and particle analysing system.** **A)** **1]** the Scireq electronic cigarette module connected to an overflow and condensation collection chamber. **2]** Pump #1 generating the puffs and pump #2 responsible for maintaining the bias flow. **3]** Particle density analyser. **4]** Output to the 45L dilution drum. **B)** **5]** Dilution drum. **6]** SMPS particle analyser.

**TABLE S1.** Statistical analysis of the impact of electronic cigarette settings and e-liquid constituents on particle size distribution

**TABLE S2.** Statistical analysis of the impact of electronic cigarette settings and e-liquid constituents on predicted lung deposition of aerosolized particles
